# Evolution of SARS-CoV-2: BA.4/BA.5 Variants Continues to Pose New Challenges

**DOI:** 10.3390/v14122610

**Published:** 2022-11-23

**Authors:** Neha Quadir, Jasdeep Singh, Anwar Alam, Asrar Ahmad Malik, Syed Asad Rahman, Subhash Hira, Nasreen Zafar Ehtesham, Durai Sundar, Seyed Ehtesham Hasnain

**Affiliations:** 1 ICMR-National Institute of Pathology, Safdarjung Hospital Campus, New Delhi 110029, India; 2 Department of Biochemical Engineering and Biotechnology, Indian Institute of Technology-Delhi, New Delhi 110029, India; 3 Department of Life Science, School of Basic Sciences and Research, Sharda University, Greater Noida 201310, India; 4 BioInception Pvt. Ltd., Swift House Ground Floor, 18 Hoffmanns Way, Chelmsford, Essex CM1 1GU, UK; 5 Department of Global Health, University of Washington, Seattle, WA 98195, USA

**Keywords:** SARS-CoV-2, BA.4/BA.5, L452R, F486V

## Abstract

The acquisition of a high number of mutations, notably, the gain of two mutations L452R and F486V in RBD, and the ability to evade vaccine/natural infection-induced immunity suggests that Omicron is continuing to use “immune-escape potential” as an evolutionary space to maintain a selection advantage within the population. Despite the low hospitalizations and lower death rate, the surges by these variants may offset public health measures and disrupt health care facilities as seen recently in Portugal and the USA. Interestingly these BA.4/BA.5 variants have been found to be more severe than the earlier-emerged Omicron variants. We believe that aggressive COVID-19 surveillance using affordable testing strategies might actually help understand the evolution and transmission pattern of new variants. The sudden dip in reporting of new cases in some of the low- and middle-income countries is an alarming situation and needs to be addressed as this could lead to undetected transmission of future variants of interest/concern of SARS-CoV-2 in large population settings, including advent of a ‘super’ virus. It would be interesting to examine the possible role/influence, if any, of the two different kinds of vaccines, the spike protein-based versus the inactivated whole virus, in the evolution of BA.4/BA.5.

## Main Text

**Figure 1:**
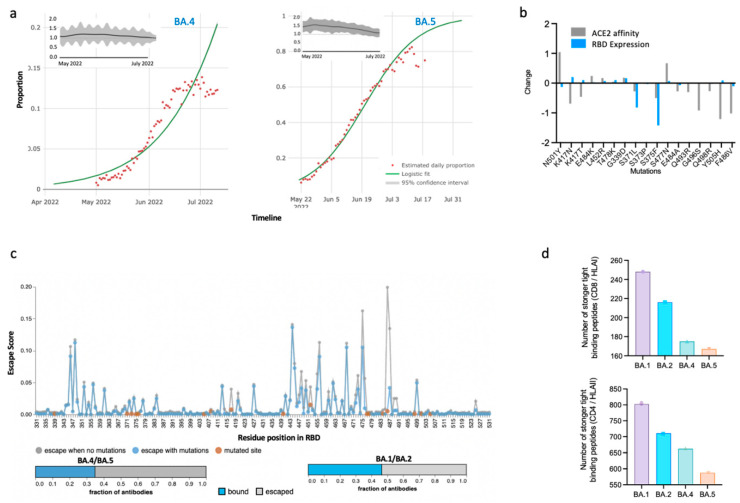
Characteristics of Omicron BA.4 and BA.5 variants. (**a**) Relative growth advantage of the BA.4 and BA.5 variant obtained by the logistic fitting of the proportion of SARS-CoV-2 variant sequences globally. The relative growth advantage estimates are based on the proportion of the specific variant (BA.*)-positive sequences over a period of time as compared to cocirculating strains. The model assumes that the current advantage is due to a transmission advantage, which is contributed to by the immune evasion capabilities, transmission characteristics, or prolonged infection time. Adapted from Chen C et al. [4]. The insets depict temporal variations in the current reproduction number along the emergence of BA.4 or BA.5 variants. (**b**) Effects of RBD mutations on ACE2 binding affinity (gray) and RBD expression in S protein. Adapted from Starr TN et al. [12]. (**c**) Prediction of immune escape and polyclonal antibody evasion upon mutations in ancestral SARS-CoV-2. Orange dots indicate mutations in RBD. The horizontal stacked bar graph shows the fraction of polyclonal antibodies which can bind or escape from the BA.4/5 omicron variant (Left) and BA.1/2 variants (Right). Adapted using “Escape calculator for SARS-CoV-2 RBD” [13]. (**d**) Number of strongly tight binding peptides to HLA 1 and HLA II in the BA.1, BA.2, BA.3, and BA.5 variants. Adapted from Nersisyan et al. [14].

The SARS-CoV-2 virus is not resting. The diversification of SARS-CoV-2 Omicron (BA.1 and BA.2) variants of concern (VoC) to BA.4 and BA.5 is posing new challenges [1]. These fast-spreading new variants have now become the most dominant strains globally. Intriguingly, the BA.4/BA.5 variants and the Delta variants exhibit similar mutation, ‘L452R’. PCR-based tests that relied on L452R-based detection [2] could have led to the misdiagnosis of BA.4/BA.5 during their earlier emergence. Moreover, recent reports suggest that BA.4/BA.5 pose a greater risk to health than BA.1/BA.2 [3]. Corresponding modelling studies indicate a 40% (95%CI: 39–40%) and 86% (95%CI: 85–87%) growth advantage for BA.4 and BA.5 variants, respectively [4], implying that these variants can replace preceding variants such as BA.2. The effective reproduction number (Re) based upon modelling estimates can remain more than 1 for BA.4/BA.5 variants, indicating its increased transmissibility within the population (reproduction number less than 1 indicates a decline in viral spread) [5] (Figure 1a). Interestingly, the BA.4/BA.5 showed substantial escape from neutralizing antibodies in both vaccinated (booster dose included) and naturally (with BA.1 or BA.2) infected individuals [1]. SARS-CoV-2 is seemingly under tremendous evolution, with novel mutations emerging very rapidly that are affecting disease severity and influencing host–pathogen interactions [6,7]. These mutations are evolutionary adaptations for viral fitness of the SARS-CoV-2 to adapt to the changing immune profile of the population. For example, the accumulation of multiple mutations (Figure 1b) in SARS-CoV-2 VoC, including Alpha, Beta, Gamma, Delta, and Omicron (BA.1), has resulted in higher host receptor ACE2 affinity and which has influenced their transmission behavior by increasing the transmissibility [8,9]. Other studies have highlighted that these mutations may also increase host receptor scanning by regulating the opening behavior of the RBD(s) of S proteins [7,10]. Hence, it has become imperative to identify the mutational hotspots that are pertinent to the further divergence of viral strains [11].

Compared to BA.1/BA.2, the BA.4 and BA.5 variants of SARS-CoV-2 show a distinct presence of the L452R and F486V mutations in the receptor binding domain (RBD) of their Spike (S) protein and additional V3G (S protein) and T481M (nonstructural protein 13) mutations in the BA.5 variant. The presence of S protein mutations, which can reduce host ACE2 receptor affinity and expression of RBDs (Figure 1b) [12,15] is concurrent with experimentally determined similar RBD–ACE2 binding affinities for BA.1/BA.2 and BA.4/BA.5 variants [16,17]. Despite the comparable ACE2 affinity of S protein of BA.4/BA.5, it has the extraordinary ability to evade vaccine/natural infection-induced immunity. This clearly suggests that Omicron is continuing to use “immune-escape potential” as a priority evolutionary space to maintain a selection advantage within a population. This is in-sync with the computationally calculated potential of BA.4/BA.5 to escape a higher proportion of RBD-neutralizing antibodies compared to that of the BA.1 and BA.2 omicron variants (Figure 1c). This increase in the proportion of escaping neutralizing antibodies is attributable to two key mutations, L452R and F486V, which were also shown to confer reduced neutralization by sera from triple-vaccinated individuals compared to BA.1 and BA.2 [17]. 

L452R, has been particularly seen in various lineages, specifically the Delta, B.1.427, and B.1.429 emerging by acquisition of this mutation, suggesting a strong positive selection of this mutation [18]. Furthermore, because it is located near the ACE2-interacting interface of RBD, this mutation has been linked to a strong binding with hACE2 as well as an altered ability of neutralising antibodies to bind these spike mutant variants, resulting in greater transmissibility and immune escape potential [19,20]. This mutation also enhances the fusogenicity of the virus, which eventually culminates in enhanced viral uptake and replication [13]. Further, L452R, which is also responsible for the escape from HLA-A24-mediated cellular immunity, poses a new threat by modulating the evolution of viral phenotypes [21]. SARS-CoV-2 has the interesting property of fusing the nearby cells to form multinucleated cells called syncytia that help viruses to spread and evade the immune system. A pseudo-Omicron virus containing L452R showed enhanced S1/S2 cleavage, which is directly related to the formation of syncytia upon infecting Huh-7, 293T-ACE2, and H1299-ACE2 cells. Moreover, lactate formation has been found to be enhanced in all the three cell types upon infection with Delta and L452R-positive Omicron [22], revealing metabolic reprogramming and exhibiting an enhanced glycolysis known as ‘Warburg effect’. This enhanced glycolysis might be used by Delta or is being used by BA.4/BA.5 to support increased infectivity. The F486 substitution(s), which have been observed in immune-compromised individuals also act as sites to confer antibody escape and binding sensitivity to RBD-directed monoclonal antibodies [17].

Despite the notion in certain sections of the scientific community that immune responses mounted by natural infection may excuse certain individuals from mandatory vaccination [23], the reinfection caused by BA.4 and BA.5 variants in previously infected BA.1/BA.2 individuals suggests otherwise [24], indicating the role of the evasion of neutralizing antibodies within potentially all the Omicron variants [16]. Precomputed analyses of effects of SARS-CoV-2 mutations on CD8 and CD4 T-cell epitopes showed a reduction in the number of strong tight-binding viral peptides to Class I and Class II HLA peptides in BA.4/BA.5 compared to BA.1/BA.2 (Figure 1c) [14]. Specifically, the number of strong tight-binding peptides was reduced to three CD8 (HLA I) alleles—HLA-A*30:01, HLA-A*31:01, and HLA-A*68:01. These observations indicate that the accumulation of further new mutations in BA.4/5 variants can alter diseases’ susceptibility and severity within the population. 

The global vaccination has undoubtedly played a crucial role in limiting the morbidity and mortality associated with COVID-19. Considering the severity of the pandemic, several types of vaccines were identified and distributed in a short span of time. However, there are concerns among the scientific community regarding the possibility of the rise of variants of concern due to the low or nonjudicious use of vaccinations that could be breeding grounds for vaccine-evading variants. Research suggests that a third dose of vaccine enhances the antibody response and lowers the likelihood of mild infections, whereas the mild infections are highly capable of creating variants that may be vaccine nonresponsive in persons who have only two prior doses [25]. Several studies have reported on the waning effect of vaccines with the passage of time. Moreover, the vaccine-induced neutralizing ability was observed to be reduced significantly (BBV152 Covaxin/Covishield used in settings in India) against the omicron variant [26]. The stress to survive in individuals immunized with different vaccines may induce a viral strain to evolve differently. These observations warrant an in-depth study on the effect of using different types of vaccines in the population and the role they might play in the emergence of aggressive variants.

It may also be interesting to note that this diversification of Omicron (with mutations of concern/interest) may further aggravate immunological and epidemiological concerns. Moreover, evolutionary geneticists believe that it is imperative to differentiate BA.4/BA.5 variants from BA.2 variants in public databases to avoid any kind of misunderstanding. It is speculated that such misunderstandings may trivialize the mutational diversity of new variants [27]. Currently, it is clear that BA.4/BA.5 have used ‘immune evasion’ as an evolutionary space to gain fitness potential among the population. Given the potential role of the L452R mutation in mediating resistance to monoclonal antibodies, facilitating T-cell immunity escape, and enhancing spike fusogenicity, its prevalence should be closely monitored through meticulous sequencing or developing variant-specific diagnostic assays. A recent modelling study has aptly estimated that providing the vaccine and antiviral therapies to vulnerable populations and persistent use of nonpharmaceutical intervention is likely to prevent overburdening of the health care system in future waves of Omicron variants [28]. The periodic waves of Omicron variants seem to be more or less predictable with waves correlating with a dip in population wide immunity, but emphasis needs to be placed on timely prediction of the outbreaks of novel variants from distant branches on the SARS-CoV-2 family tree. Nonetheless, the descendant of BA.4, BA.4.6 has arrived with two additional sets of mutations—R346T and N658S. These two mutations have recently been shown to provide resistance against monoclonal antibody therapies which further suggests the vicious rise of BA.4 and BA.5 variants with immune evasion as a possible evolutionary strategy [29].

The following can be concluded: (1) Contrary to the earlier notion that SARS-CoV-2 S protein variants confer a transmission advantage via modulating ACE2 affinity/RBD expression, the newer variants can maintain a selection advantage in addition to immune-evasion at the expense of reduced or similar ACE2 affinity. (2) Aggressive COVID-19 surveillance using affordable testing strategies might actually help understand the evolution and transmission pattern of new variants. (3) The sudden dip in the reporting of new cases in some of the low- and middle-income countries is an alarming situation and needs to be addressed, as this could lead to undetected transmission of future variants of interest/concern of SARS-CoV-2 in large population settings, including the advent of a ‘super’ virus. (4) It would be interesting to examine the possible role/influence, if any, of the two different kinds of vaccines, the spike protein-based vs. inactivated whole virus in the evolution of such ‘aggressive’ BA.4/BA.5 variants of concern. Finally, (5) the more fundamental issue of the immunological, medical, and other consequences of global mass vaccination with vaccines essentially approved for emergency use on the human host deserves to be given greater attention.
